# Untangling the taxonomy of dahlia mosaic virus

**DOI:** 10.1007/s00705-022-05567-8

**Published:** 2022-08-15

**Authors:** Andrew D. W. Geering, Alistair R. McTaggart, Pierre-Yves Teycheney

**Affiliations:** 1 Queensland Alliance for Agriculture and Food Innovation, The University of Queensland, St Lucia, QLD 4072 Australia; 2 CIRAD, UMR PVBMT, 97410 Saint Pierre, La Réunion France

## Abstract

In this brief note, we review the taxonomic history of dahlia mosaic virus (DMV) and related viruses. DMV is the only officially recognized caulimovirus known to infect dahlia (*Dahlia variabilis*) plants, although this virus appears to be relatively rare as a pathogen compared to a more recently described but unclassified caulimovirus called dahlia common mosaic virus (DCMV). We have undertaken a new set of analyses to test the hypothesis that DCMV represents a new caulimovirus species whose members infect dahlia, but we ultimately reject this hypothesis. A probable sequencing error was identified in the reference genome sequence of DMV, and consequently, we recommend that an alternative virus isolate be nominated as the exemplar for this species. In accordance with the new binomial nomenclatural system, it is proposed that the virus species be called “*Caulimovirus dahliae*”.

Dahlia mosaic is one of the most prevalent and damaging viral diseases of dahlia (*Dahlia variabilis*) in Europe and the USA [4, 19]. Dahlia mosaic virus (DMV), the putative causative agent of the disease, was one of the first officially recognized members of the genus *Caulimovirus* (initially called the ‘cauliflower mosaic virus group’), appearing in the first report of the International Committee on Nomenclature of Viruses, published in 1971 [23]. At this early point in the development of a classification scheme for viruses, formal taxonomic proposals were not required to recognise a new virus species. Nevertheless, there was strong evidence at the time to support the classification of DMV and cauliflower mosaic virus (CaMV) as closely related but distinct members of this virus genus. The characters that were used for classification included the size and shape of the virion, the presence of electron-dense inclusion bodies in the cytoplasm of infected cells, the mode of transmission, differences in host range, and weak serological relatedness of the viruses in gel diffusion tests [3, 4].

The diagnostic criteria used to distinguish DMV from CaMV are still relevant today, although they have been supplanted by nucleotide-sequence-based criteria to differentiate between related viruses that cause similar disease symptoms and share many other biological characteristics. Genome architecture and phylogeny are now used for classification to the taxonomic rank of genus, and the principal criterion used to demarcate different virus species in the family *Caulimoviridae* is less than 80% nucleotide sequence identity in the conserved reverse transcriptase and ribonuclease H coding domains of the polymerase gene (open reading frame [ORF] 5) [22].

Nicolaisen [14] provided the first sequence data for dahlia mosaic virus (DMV), obtained from a 1195-bp PCR amplicon from a dahlia plant with mosaic symptoms, which was obtained from the Dutch Flowerbulb Inspection Service. This DNA sequence, deposited in the GenBank database on 13 November 2002 under INSDC accession code AJ515906.1, contains parts of ORF 4 (capsid protein) and ORF 5 (polymerase protein).

Almost contemporaneously with the publication by Nicolaisen [14], IB Maiti and S Pattanaik deposited six sequences representing a nearly complete genome sequence of DMV in the GenBank database on 5 May 2003 (INSDC accession codes AY309479.1, AY309480.1, AY291585.1, AY291586.1, AY291587.1, and AY291588.1). These unpublished sequence records of the genome of DMV are problematic for a stable taxonomy of DMV. Firstly, metadata describing the origin of the sequences were not provided. Secondly, sequences of each open reading frame (ORF) were deposited as individual flat files and not as a contiguous genome sequence. Finally, the intergenic region containing the 35S promoter sequence was excluded from the series of sequence depositions.

A complete genome sequence of DMV was later deposited in GenBank by IB Maiti and S Pattanaik on 15 September 2015, which derived from an infected dahlia plant collected on 20 May 1980 in Portland, Oregon (INSDC accession code JX272320.1). This virus isolate has been nominated the exemplar for DMV by the International Committee on Taxonomy of Viruses (ICTV), and accordingly, the genome sequence is recognized as the reference sequence for DMV by the NCBI (RefSeq accession code NC_018616.1). The citations associated with INSDC accession code JX272320.1 were Banerjee et al. [2] and Sahoo et al. [20], but both publications describe characterisation of the 35S promoter and do not include details of the origin of the virus isolate or methods used to generate the sequence, nor do they describe essential features of the virus genome, including its episomal nature.

Pahalawatta et al. [15], Pahalawatta et al. [16], and Pappu et al. [18] described two more distinct caulimoviruses from dahlia: dahlia common mosaic virus (DCMV) and DMV-D10. In these papers, the virus from the Netherlands [14] was labelled as DMV-Holland, and that sequenced by Maiti and Pattanaik, DMV-Portland. DMV-Holland and DCMV were most alike, having 90% nucleotide (nt) sequence identity in the overlapping region of the genome sequence [18]. However, Pappu et al. [18] used DMV-Portland as the exemplar for DMV because a near-complete genome sequence was available, which allowed for more comprehensive pairwise sequence comparisons and phylogenetic analyses. When ORF 5 protein sequences were compared, DCMV had only 70−71% amino acid (aa) sequence identity to both DMV-Portland and DMV-D10, which justified recognition of a new caulimovirus species. However, this species has never been officially approved by the ICTV despite its persistence as a taxon in the literature [5, 9, 24]. Pappu et al. [18] followed the precedent set by Maiti and Pattanaik and deposited the genome sequence of DCMV, minus the intergenic region, in separate parts representing each ORF (INSDC accession codes EU090952, EU090953, EU090954, EU090955, EU090956, and EU090957). Pappu et al. [18] provided the name ‘DMV-Holland’ instead of DCMV in the sequence definition field, even though the authors had concluded they had sequenced a member of a distinct virus species.

DMV-D10 is quite enigmatic, as it has an aberrant genome architecture compared to all other caulimoviruses [16]. Specifically, ORF 3, which encodes an aphid transmission helper protein, is absent in DMV-D10, and the putative capsid protein also contains a C-terminal deletion and is fused in-frame with the polymerase protein. As a result of these large deletions, the genome of DMV-D10, at 7 kbp, is much shorter than a normal caulimovirus genome, which is about 8 kbp [22]. Southern blot hybridization and fluorescent *in situ* hybridization experiments suggest that DMV-D10 is integrated in the nuclear genome of dahlia [7, 15], and it is highly unlikely that this endogenous viral element is able to replicate autonomously, as it is missing the conserved zinc finger motif in the nucleocapsid domain of the capsid protein that is essential for infectivity of the virus [8]. However, the replication of DMV-D10 might be supported by a coinfecting virus such as DCMV. DMV-D10, renamed Dahlia variabilis endogenous pararetroviral sequence (DvEPRS) by Eid and Pappu [7], was detected in wild plants of four different *Dahlia* species (*D. coccinea*, *D. rupicola*, *D. tenuicaulis* and *D. sherffii*) growing in their natural habitats in west-central Mexico, suggesting that the integration events occurred well before the domestication of dahlia [6].

In recent years, the genomes of many more representatives of DMV, DCMV, and DMV-D10 from around the world have been sequenced in their entirety or in part, providing a much better foundation to resolve the classification of these viruses. To test the hypothesis that DCMV is a member of a distinct virus species, we have undertaken a new analysis using the complete data set available to us in April 2022. The ORF 5 protein sequences of the exemplar isolates of all officially recognized or tentative caulimovirus species, as well as DMV, DCMV, and DMV-D10, were downloaded from the NCBI Protein database and aligned using COBALT [17]. This protein alignment was then used to guide alignment of the corresponding DNA sequences using the TranslatorX server available at http://161.111.161.41/index_v4.html [1]. For reasons that are explained below, the ORF 5 sequence of DMV-Portland (JX272320.1) was added to the existing DNA alignment using the ‘realign selected sequences’ function in CLUSTAL X v. 2.1. For the phylogenetic analysis, the variable 5’ and 3’ ends of the alignment were deleted so that the alignment only represented the conserved aspartic protease (cd00303), reverse transcriptase (cd00304), and ribonuclease H1 (pfam17917) domains. This alignment was further truncated to the reverse transcriptase (RH) and ribonuclease H1 (RH1) domains (nt 4450–5661 of NC_001497.2) for pairwise sequence comparisons using MEGA v. 11 [21]. A phylogenetic tree was inferred by maximum likelihood using IQ-Tree v2 with an automatic substitution model (command -m TEST) and 10,000 replicates each of an approximate likelihood ratio test and ultrafast bootstraps [12, 13]. Relationships among the sampled populations of DCMV and DMV-D10 were visualised with SplitsTree v4 [10], and the pairwise homoplasy index was calculated as a measure of recombination within each population.

Our phylogenetic analysis identified DMV as sister to DCMV (100% support from UltraFast Bootstraps and an approximate likelihood ratio test, Fig. 1A). The sequenced isolates of DCMV have low genetic diversity (98.8–99.5% nt sequence identity in the RT and RH1 coding domains) and a signature of clonal reproduction based on a lack of reticulate branches in SplitsTree (Fig. 1B), and a high pairwise homoplasy index (≥ 0.5 significant for clonal reproduction). These results suggest that DCMV has spread around the world by trade of infected vegetative propagules (rooted cuttings or root tuber pieces) of one or a small number of dahlia cultivars. Within the conserved RT and RH1 coding domains of ORF 5, DMV-Portland and the six isolates of DCMV have 85.5–85.9% nt sequence identity, which would suggest that these viruses are conspecific. However, DMV-Portland and DCMV also share 84.0% and 82.5–82.8% nt sequence identity, respectively, with mirabilis mosaic virus (AF454635), which brings into question the universal applicability of the 80% nt identity threshold for caulimovirus species demarcation. Clearly, more sampling of the caulimovirus sequence populations in dahlia and *Mirabilis jalapa* is required to more accurately define the species boundaries and to determine if MiMV is also synonymous with DMV.

**Fig. 1:**
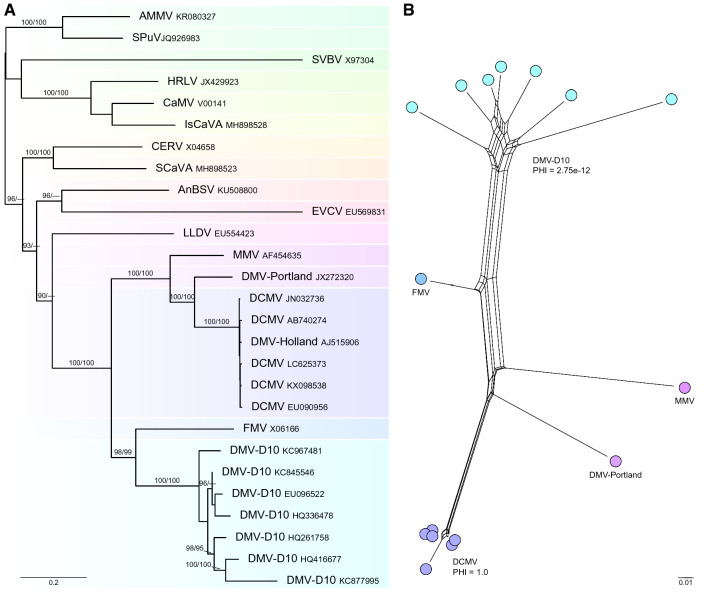
Phylogenetic analysis of the genus *Caulimovirus* based on alignment of aspartic protease, reverse transcriptase, and ribonuclease H1 coding sequences. (A) Phylogram from a maximum-likelihood search in IQTree v2, with support values above nodes from 10,000 replicates each of UltraFast Bootstraps (≥ 95%) and approximate likelihood ratio tests (≥ 90%). (B) SplitsTree analysis showing all relationships among data. Reticulation is interpreted as recombination or homoplasy. Pairwise homoplasy index (PHI) calculated for DCMV and DMV-D10 (< 0.05 is significant for recombination). AMMV, atractylodes mild mottle virus; AnBSV, angelica bushy stunt virus; CERV, carnation etched ring virus; DCMV, dahlia common mosaic virus; DMV, dahlia mosaic virus; EVCV, eupatorium vein clearing virus; FMV, figwort mosaic virus; HRLV, horseradish latent virus; LLDV, lamium leaf distortion virus; MMV, mirabilis mosaic virus; SiCaAV, silene caulimovirus A virus; SPuV, soybean Putnam virus; SVBV, strawberry vein banding virus. INSDC accession codes for the sequences used in the analyses are provided to the right of the virus acronyms

Pappu et al. [18] obtained a significantly more divergent amino acid (aa) sequence identity figure for the aligned polymerase protein sequences of DMV-Portland and DCMV than would be expected from our pairwise nucleotide sequence comparisons. This result can be explained by a single nucleotide deletion in ORF 5 of DMV-Portland (position 5070 in NC_018616.1) that both alters the reading frame of the sequence and causes premature termination of the ORF, leading to a large deletion at the C-terminus of the RH1 enzyme, including much of the catalytic domain [11]. If the DNA sequence downstream of the putative indel in DMV-Portland is excluded in sequence comparisons, then the conceptually translated RT and RH1 domains have 93.4% aa sequence identity. We hypothesise that the indel in the DMV-Portland genome sequence is a sequencing error, as otherwise it would be predicted to render the virus replication-defective due to deletion of several sequence motifs that are conserved across all ribonuclease H1 proteins classified in protein family pfam17917. Alternatively, it is possible that that the sequence is accurate but corresponds to a mutated endogenous caulimoviral element.

DCMV appears to be a clonal lineage of DMV, and following the current ICTV species demarcation criteria, there is no justification to recognise two different virus species. The debate as to which name to apply to the taxon has been made redundant with the introduction of Latinized binomial species names [25], as an entirely new taxon name will need to be created for the caulimovirus associated with dahlia mosaic disease. We propose that this virus species be called “*Caulimovirus dahliae*” and that the exemplar isolate for this species be DCMV isolate JP (INSDC accession code LC625373), as this is the only virus isolate for which Koch’s postulates of pathogenicity have been satisfied through use of an infectious clone [24]. We also recommend use of the name “dahlia common mosaic virus” when referring to the virus in a vernacular sense. While DMV-D10 is clearly distinct from DCMV, replication-defective endogenous caulimoviral elements are not yet formally classified by the ICTV, and therefore, there is not a pressing need to introduce a new taxon name.
